# Implementing a Blood Pressure Measurement Curriculum for First-Year Medical Students

**DOI:** 10.1007/s40670-023-01825-9

**Published:** 2023-07-06

**Authors:** Margaret Fisher, Ariel Harris, Thomas Koonce, Gary Beck Dallaghan, Catherine L. Coe

**Affiliations:** 1 University of North Carolina School of Medicine, Chapel Hill, NC USA; 2 Department of Family Medicine, University of North Carolina School of Medicine, Chapel Hill, NC USA; 3 University of Texas at Tyler School of Medicine, Tyler, TX USA

**Keywords:** Medical education, Blood pressure, Hypertension, Curriculum

## Abstract

A core clinical skill medical students need to learn is obtaining an accurate blood pressure (BP) reading. We developed a standardized BP curriculum for first-year medical students. Medical students completed online modules and a hands-on skills session to learn BP skills. Pre- and post-surveys and an observed structured clinical encounter (OSCE) assessed student confidence and ability to accurately measure BP. Student confidence and mean OSCE scores (pre = 2.63, post = 6.51; *p* < 0.001) improved upon completion of the curriculum. The curriculum was feasible, well received, and improved student’s skills for taking an accurate BP.

## Background

Hypertension impacts approximately half of the US population, with a disproportionate impact on underrepresented minorities [1–4]. Poorly controlled blood pressure (BP) causes significant morbidity and mortality resulting in heart attacks, strokes, and heart failure [5, 6]. Accurate BP measurement in the healthcare setting is an essential step in improving BP control.

While measuring BP is a common procedure, student training in BP measurement is often inadequate and varies widely between schools. In one study of 159 students from medical schools nationwide, only one student demonstrated proficiency in measuring BP [7]. To address gaps in education of this essential skill, a standardized longitudinal program combining e-learning and hands-on training was integrated into the PatientCentered Care Course (PCC) curriculum at the University of North Carolina School of Medicine (UNC SOM). The project aimed to (1) implement the American Medical Association (AMA) BP Measurement Essentials Modules for medical trainees [8]; (2) expose students to the disparities in healthcare related to hypertension; and (3) pair BP training with training in clinical reasoning and management of chronic diseases through patient cases related to hypertension.

## Activity

**Fig. 1:**
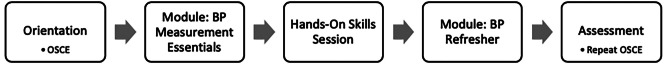
Blood pressure curriculum schematic. OSCE, observed structured clinical encounter; BP, blood pressure

**Fig. 2:**
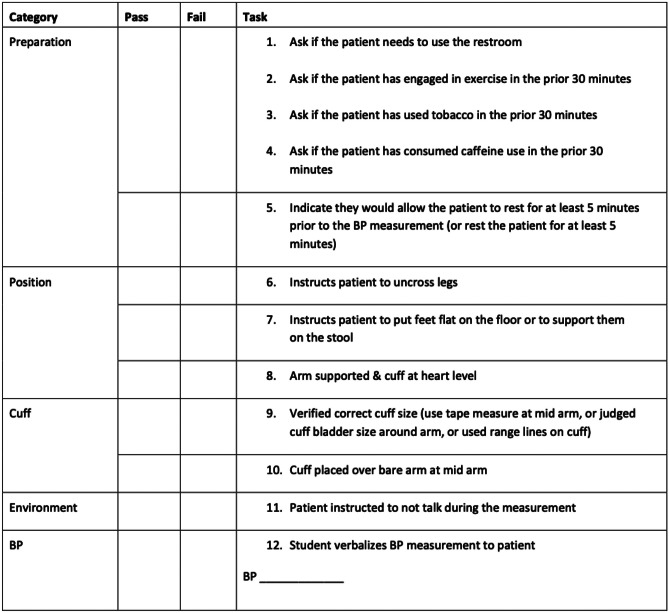
Standardized patient rubric. Modified from supplemental material from Hayer et al.

This study was deemed exempt by the UNC Institutional Review Board (IRB #21–1744). All medical students at the UNC SOM are enrolled in the three-semester PCC course designed to teach basic clinical skills prior to entering clinical rotations. As part of the PCC course, 190 first-year medical students completed the longitudinal BP curriculum described in Fig. 1. During new student orientation, students completed a baseline observed structured clinical examination (OSCE) where standardized patients scored them on a scale of zero to twelve based on completion of twelve tasks necessary to obtain an accurate BP measurement (Fig. 2) [9]. BP accuracy was also recorded. Trained clinicians measured standardized patients’ BP prior to student encounters. If the students’ obtained BP differed by less than 5% from the baseline BP taken, it was considered accurate. This threshold was based on the principle that BP variability is a normal physiologic phenomenon [10]. In a real clinical setting, BP measurements vary between consecutive visits by an average of 12 mmHg (approximately 10% in patients with normal systolic BPs) [11]. Because there was only a short time interval between baseline and student measurement, we assumed the variability would be considerably low, and set an arbitrary threshold of 5%.

Students then completed the online AMA BP Measurement Essentials: Student Edition module [8]. The module, which had an estimated completion time of 40 min, explained the importance of accurate BP measurement, reviewed current epidemiology, illustrated basic concepts and terminology related to BP measurement, presented different equipment used to measure BP, demonstrated how to properly prepare and position a patient, and explained how to perform BP measurements on various BP devices. Module completion was tracked by having students upload completion certificates to the school’s online learning management system.

Midway through the first semester, students took part in a hands-on skills session. The session began with a brief lecture to review BP measurement material. Students practiced measuring BP at stations with manual and semi-automatic devices.

During the second semester, approximately 6 months following the initial module and hands-on skills session, students were asked to complete the online AMA BP Refresher module [12]. The module reviewed concepts covered in the initial BP measurement essentials module including preparing and positioning patients and accurately obtaining BP measurements.

To further address the aims of the project, students participated in two supplemental activities. During the cardiology unit, students attended a lecture on disparities in cardiovascular disease which included hypertension. This was a component of a longitudinal lecture series which highlighted disparities in various illnesses as well as themes of social justice. Students also participated in a case-based learning exercise in their PCC small-group meetings aimed at developing clinical reasoning skills. The case involved a patient with recently diagnosed hypertension undergoing workup and medication adjustments over the course of two clinic visits.

Student evaluation was two-fold. Students completed pre- and post-surveys rating their current level of confidence in their ability to perform the following: properly prepare and position the patient prior to measuring BP, choose the correct BP cuff size, accurately measure BP using manual methods, and accurately measure BP using automated methods. Secondly, students repeated the OSCE and were again scored based on the same twelve tasks, as well as BP accuracy.

IBM SPSS Statistics version 28.0 was used for data analysis. Descriptive statistics were calculated where appropriate. Crosstabulations with McNemar test were used to compare pre- and post-curriculum OSCE scores and BP accuracy.

## Results

### Confidence Levels

A summary of self-reported confidence levels is summarized in Table 1. At baseline, 121 students out of 182 total students (66.5%) were either *very confident* or *mostly confident* in their ability to properly prepare and position the patient prior to measuring BP compared to 129 students out of 151 total students (85.4%) after completing the curriculum. One hundred one students out of 179 total (55.5%) at baseline versus 120 students out of 150 total (79.5%) post-curriculum were either *very confident* or *mostly confident* in their ability to choose the correct BP cuff. Eighty students out of 181 total (43.9%) and 129 students out of 182 total (70.9%) at baseline versus 124 out of 151 total (82.1%) and 134 out of 150 total (88.8%) post-curriculum were either *very confident* or *mostly confident* in their ability to accurately measure BP using manual methods and automated methods, respectively.

**Table 1:** Student-rated confidence levels, OSCE score, and BP accuracy before and after curriculum completion

	**Pre-module (*N* (%))**	**Post-module (*N* (%))**	
**Confidence levels**			
**Properly preparing and positioning patient**			
Very confident	64 (35.2)^a^	87 (57.6)^a^	
Mostly confident	57 (31.3)	42 (27.8)	
Somewhat confident	35 (19.2)	12 (7.9)	
Slightly confident	17 (9.3)	10 (6.6)	
Not at all confident	9 (4.9)	0 (0)	
**Choose the correct blood pressure cuff size**			
Very confident	36 (19.8)	74 (49.0)^a^	
Mostly confident	65 (35.7)^a^	46 (30.5)	
Somewhat confident	48 (26.4)	20 (13.2)	
Slightly confident	22 (12.1)	9 (6.0)	
Not at all confident	8 (4.4)	1 (.7)	
**Accurately measure blood pressure using manual methods**			
Very confident	25 (13.7)	66 (43.7)^a^	
Mostly confident	55 (30.2)^a^	58 (38.4)	
Somewhat confident	49 (26.9)	14 (9.3)	
Slightly confident	34 (18.7)	11 (7.3)	
Not at all confident	18 (9.9)	2 (1.3)	
**Accurately measure blood pressure using automated methods**			
Very confident	76 (41.8)^a^	94 (62.3)^a^	
Mostly confident	53 (29.1)	40 (26.5)	
Somewhat confident	31 (17.0)	10 (6.6)	
Slightly confident	14 (7.7)	6 (4.0)	
Not at all confident	8 (4.4)	0 (0)	
	**Pre-curriculum**	**Post-curriculum**	***P*-value**
**OSCE score (out of 12)**	2.63	6.51	** < 0.001**
	**Pre-curriculum (% of students)**	**Post-curriculum (% of students)**	***P*-value**
**BP accuracy**^**b**^			
Systolic BP < 0.05 difference	31.9	42.5	0.097
Diastolic BP < 0.05 difference	36.0	26.1	0.109

^a^indicates most frequently selected response for each question

^b^only students who had both pre- and post-data were included in analysis

### OSCE Scores

The mean baseline OSCE score was 2.63 out of 12 (Table 1). Scores ranged from 0 to 7. The mean score received during the repeat OSCE was 6.51 out of 12 (*p* < 0.001), ranging from 2 to 11 (Table 1). No student scored a 9 or higher at the baseline OSCE, compared to 55 students (32.0%) during the repeat OSCE.

### BP Accuracy

Results in BP accuracy are summarized in Table 1. During the baseline OSCE, 31.9% of students (36 out of 113 total) accurately recorded systolic pressures, and 36.0% (40 out of 111 total) accurately recorded diastolic pressures. After completion of the curriculum, 42.5% (48 out of 113 total) and 26.1% (29 out of 111 total) of students accurately recorded systolic and diastolic pressures, respectively. The proportion of students who recorded BP accurately did not improve significantly after curriculum completion (systolic *p* = 0.097; diastolic *p* = 0.109). Amongst those who completed the AMA BP Refresher module, 56.1% (64 out of 114 total) accurately recorded systolic BP.

## Discussion

The implementation of a standardized BP measurement module and hands-on skills session was well received by students. Out of 182 students who took a post-curriculum satisfaction survey, 136 (75%) were either extremely or somewhat satisfied. Overall student-rated confidence level increased in all measured areas: ability to prepare and position the patient prior to measuring BP, choose the correct BP cuff, and accurately measure manual and automated BP (see Table 1). OSCE scores improved significantly following completion of the curriculum (see Table 1). When looking at pre-curriculum individual-level OSCE data, points were largely earned as a result of being able to put the BP cuff on correctly, something most students have encountered either in prior clinical experience or as a patient. Post-curriculum, most students were able to complete items #6–11 on the OSCE scoresheet (instruct patients to uncross legs and place feet flat on floor, support arm at heart level, verify correct cuff size, place cuff over bare arm, and instruct patient to not talk during measurement), accounting for the increase in mean score. On average, most students failed to completely prepare the patient prior to BP measurement (i.e., ask about going to the restroom, exercise, tobacco, and caffeine) indicating a knowledge gap that should be emphasized in further teaching sessions.

While student confidence in obtaining an accurate BP improved, actual accuracy, according to our set criteria, did not (see Table 1). Furthermore, accuracy in diastolic values actually decreased, although not significantly. The reason for this is unknown. In light of these results, particular attention should be paid to determining diastolic readings in future skills sessions. We maintain that measuring patient BP is a clinical skill that requires consistent practice, like drawing blood or placing a foley catheter, for example. Mastering BP measurement with an automatic monitor would likely require less practice and result in more accurate readings. However, we feel obtaining BP manually to be a necessary skill given that an automatic monitor may not always be readily available in clinical practice or calibrated accurately.

Interestingly, yet perhaps unsurprisingly, students who reported completion of the BP Refresher module were more accurately able to measure BP compared to those who did not. This finding suggests that the modules may have a meaningful impact on students’ ability to accurately obtain BP readings. Special focus on the content contained in the module in future skills sessions may help improve student skill acquisition. During the OSCE, assessment of BP accuracy was limited by using a previously obtained BP as a baseline to which we compared student-obtained BP. We disregarded any measurement bias that could have occurred due to this as we assumed there would be minimal variation in BP between readings. In the future, this can be addressed with the use of a dual head teaching stethoscope that allows for auscultation of Korotkoff sounds to ensure an accurate assessment of student measurement.

Based on informal faculty feedback, the curriculum was feasible and relatively easy to implement. To improve the ease of implementation and student learning experience in future iterations, we plan to have standardized patients review the modules ahead of the OSCE to improve interrater reliability, have students practice obtaining a manual BP on a simulated arm for practice, and utilize a trained clinician and double-headed stethoscopes to assess for BP accuracy. We aim to improve upon the current curriculum by making it both a longitudinal and interprofessional experience. During their second year, medical students will complete the AMA Self-Measured BP module which highlights the importance of self-measured BP [13]. They will also participate in a skills session with standardized patients to practice motivational interviewing and teach-back skills. The curriculum will be extended to dental, dental hygiene, pharmacy, and nursing students at our university.

In conclusion, this standardized BP curriculum improved students’ understanding of the steps necessary to properly obtain patients’ BP. We believe that equipping future healthcare providers with this skill set is the first of many steps that may help address the large burden of disease and healthcare disparities in the USA.

## References

[1] Virani SS, Alonso A, Benjamin EJ (2020). Heart disease and stroke statistics 2020 update: a report from the American Heart Association. Circulation.

[2] Whelton PK, Einhorn PT, Muntner P (2016). Research needs to improve hypertension treatment and control in African Americans. Hypertension.

[3] Bosworth HB, Dudley T, Olsen MK (2006). Racial differences in blood pressure control: potential explanatory factors. Am J Med.

[4] Downie DL, Schmid D, Plescia MG (2011). Racial disparities in blood pressure control and treatment differences in a Medicaid population, North Carolina, 2005–2006. Prev Chronic Dis.

[5] Lewington S, Clarke R, Qizilbash N, Peto R, Collins R (2002). Age-specific relevance of usual blood pressure to vascular mortality: a meta-analysis of individual data for one million adults in 61 prospective studies. Lancet.

[6] Blood Pressure Lowering Treatment Trialists’ Collaboration (2003). Effects of different blood-pressure-lowering regiments on major cardiovascular events: results of prospectively-designed overviews of randomized trials. Lancet.

[7] Rakotz MK, Townsend RR, Yang J, Alpert BS, Heneghan KA, Wynia M, Wozniak GD (2017). Medical students and measuring blood pressure: results from the American Medical Association Blood Pressure Check Challenge. J Clin Hypertens.

[8] American Medical Association Ed Hub. BP measurement essentials: student Edition. Interactive Course. 2021. https://edhub.ama-assn.org/interactive/18594970

[9] Hayer R, Kirley K, Cohen JB (2022). Using web-based training to improve accuracy of blood pressure measurement among health care professionals: a randomized trial. J Clin Hypertens.

[10] Parati G, Ochoa JE, Lombardi C, Bilo G. Assessment and management of blood-pressure variability. Nat Rev Cardiol. 2013 Mar;10(3):143–55. 10.1038/nrcardio.2013.1. Epub 2013 Feb 12. Erratum in: Nat Rev Cardiol. 2014;11(6):314. PMID: 23399972 10.1038/nrcardio.2013.1 23399972

[11] Lu Y, Linderman GC, Mahajan S, Liu Y, Huang C, Khera R, Mortazavi BJ, Spatz ES, Krumholz HM. Quantifying blood pressure visit-to-visit variability in the real-world setting: a retrospective cohort study. Circ Cardiovasc Qual Outcomes. 2023;16(4):e009258. 10.1161/CIRCOUTCOMES.122.009258. Epub 2023 Mar 8. PMID: 36883456 10.1161/CIRCOUTCOMES.122.009258 36883456

[12] American Medical Association Ed Hub. BP measurement refresher: student edition. Interactive Course. 2021. https://edhub.ama-assn.org/interactive/18594856

[13] American Medical Association Ed Hub. Self-measured blood pressure essentials: student edition. Interactive course. 2021. https://edhub.ama-assn.org/interactive/18594870

