# Correction to: Implementing a Blood Pressure Measurement Curriculum for First-Year Medical Students

**DOI:** 10.1007/s40670-023-01967-w

**Published:** 2023-12-19

**Authors:** Margaret Fisher, Ariel Harris, Thomas Koonce, Gary Beck Dallaghan, Catherine L. Coe

**Affiliations:** 1 University of North Carolina School of Medicine, Chapel Hill, NC USA; 2 Department of Family Medicine, University of North Carolina School of Medicine, Chapel Hill, NC USA; 3 University of Texas at Tyler School of Medicine, Tyler, TX USA

**Correction to: Medical Science Educator (2023) 33:841–845**

10.1007/s40670-023-01825-9

The article “Implementing a Blood Pressure Measurement Curriculum for First-Year Medical Students”, was originally published electronically on the publisher’s internet portal on 6 July 2023 without open access.

With the author(s)’ decision to opt for Open Choice the copyright of the article changed on 15 November 2023 to © The Author(s) 2023 and the article is forthwith distributed under a Creative Commons Attribution 4.0 International License, which permits use, sharing, adaptation, distribution and reproduction in any medium or format, as long as you give appropriate credit to the original author(s) and the source, provide a link to the Creative Commons licence, and indicate if changes were made. The images or other third party material in this article are included in the article’s Creative Commons licence, unless indicated otherwise in a credit line to the material. If material is not included in the article’s Creative Commons licence and your intended use is not permitted by statutory regulation or exceeds the permitted use, you will need to obtain permission directly from the copyright holder. To view a copy of this licence, visit http://creativecommons.org/licenses/by/4.0.

The original article has been corrected.

